# It's not all about small airways disease: key limitations of oscillometry interpretation

**DOI:** 10.3389/falgy.2026.1872486

**Published:** 2026-07-10

**Authors:** David A. Kaminsky

**Affiliations:** Robert Larner MD, College of Medicine, University of Vermont, Burlington, VT, United States

**Keywords:** frequency dependance of resistance, impedance, oscillometry, peripheral airways disease, small airways disease

## Abstract

An exciting aspect of oscillometry is the ability to detect small airways disease (SAD), particularly through frequency dependence of resistance, commonly measured as R5-R20. While SAD influences oscillometry measurements, it is important to realize that oscillometry is not specific for SAD. In addition, oscillometry measurements do not take into account lung volume, which can influence the measurements and make interpretation less clear. Finally, while oscillometry is very sensitive at detecting abnormalities of lung function, it is not specific for distinct lung diseases. These limitations should be considered when using oscillometry in respiratory physiology and medicine, where oscillometry provides valuable complimentary information to other lung function tests in evaluation of lung health.

## Introduction

Oscillometry holds great promise in respiratory medicine as both a diagnostic and monitoring tool. However, there are concerns about lack of harmonization between instruments as well as selection of appropriate reference values (1–3). In addition, it is important to recognize that there are also limitations related to interpretation. With regard to the topic of this symposium related to small airways disease, an important consideration is that while frequency dependence of resistance is sensitive to small airways disease, it is not specific for small airways disease alone. In addition, we will review two other limitations of oscillometry interpretation, which relate to dependence of oscillometry measurements on lung volume, and lack of disease discrimination. Recognizing these limitations is not meant to diminish the importance of oscillometry; rather, these limitations should be recognized so that oscillometry results are interpreted accurately and viewed appropriately in the clinical context of the patient's overall lung health.

### Non-Specificity of R5-R20 for small airways disease

One of the commonly cited advantages to oscillometry is the ability to measure small airways disease (SAD). Usually this is determined from an increase in frequency dependence of resistance, often measured as R5-R20, or equivalent (such as R5-R19). SAD is also thought to be indicated by more negative X5 or increase in AX. While SAD may become manifest as changes in R5-20, X5 or AX, it is important to realize that none of these parameters are specific for SAD (3). In particular, ventilation heterogeneity, which may include both small and large airways, mechanical heterogeneity of the lung parenchyma, and upper airway and airway wall shunting, may also result in abnormal R5-R20, X5 or AX.

Small airway disease is difficult to detect by conventional testing and yet appears to play an important role in disease pathogenesis and symptoms (4). While the typical definition of SAD is disease involving airways < 2 mm inner diameter, it may be more clinically relevant and useful to distinguish central airways from peripheral airways. In a practical sense, central airways may be considered those that can be seen by bronchoscopy down to the 4th generation or detected by chest CT down to the 6th generation; all airways beyond these limits may be considered peripheral airways (5). In terms of size, bronchoscopically visualized 4th generation airways are on the order of 5 mm in diameter, and CT-detected 6th generation airways are on the order of 3 mm in diameter (6), so the lung periphery by this definition would include airways < 3–5 mm in diameter, which are not all strictly small airways. While the terms “peripheral airways” and “small airways” are commonly used interchangeably (5), it is not entirely accurate to say that the lung periphery reflects only SAD.

Another consideration is how SAD is manifested physiologically. The term “functional small airways disease” might be a more accurate interpretation, since diseases of the small airways result in important physiological consequences (7). These include increased peripheral airway resistance and ventilation heterogeneity, with propensity for early airway closure and gas trapping.

How can oscillometry distinguish large vs. small airways disease? This is mainly accomplished by using imposed pressure perturbations of different frequencies. Low frequency waves (< 5 Hz) are better able to penetrate out to the lung periphery compared to higher frequency waves (> 10 Hz), as has been directly demonstrated in rabbits using an alveolar capsule technique (8). This is similar to the case of ultrasound waves and how they penetrate to different tissue depths depending on frequency (9). Higher frequency ultrasound waves (> 10 MHz) can only penetrate tissues to a limited extent because their energy is more scattered and attenuated (absorbed) by tissues, whereas lower frequency waves (< 5 MHz) penetrate more deeply. The situation is similar with the pressure waves generated by oscillometry, however much lower frequencies (< 40 Hz – not “mega” Hz) than used in ultrasound are needed to provide physiologically relevant information about the respiratory system.

The lowest frequency that can be realistically used needs to be at least an order of magnitude greater than the natural breathing frequency to avoid excess noise in the signal from harmonics (10). As the natural resting breathing frequency is 0.2–0.3 Hz (12–18 breaths/minute), this sets the lowest frequency in the 2–3 Hz range. However, using a minimal frequency of 2–3 Hz in spontaneously breathing individuals is technically challenging due to poor signal-to-noise characteristics (11). The typical commercial oscillometry devices employ the range of 5–40 Hz.

In addition to scatter and attenuation, it may be useful to think about how frequency determines depth of detection in terms of time constants. The time constant for a pressure wave is the time it takes the wave pressure to decay to 1/*e* (∼37%) of its starting value. To maximally transmit energy and record the resulting return of energy, measured as impedance by oscillometry, it is optimal to use a wave signal that approximates the time constant of the entire respiratory system, which is about 0.2 s This corresponds to a frequency of ∼1 Hz. This frequency would allow the wave to have time to transmit its energy to the lung and be modified by local lung mechanics before it reflects back to the sensor at the airway opening (12). In general, frequencies < 5 Hz reflect peripheral lung mechanics in both adults (13) and children (14). Frequencies < 2 Hz are needed to detect tissue viscoelasticity (15, 16). Computational modeling has shown that, depending on the degree of mean constriction and heterogeneity of constriction, the conducting airways account for most of input impedance at frequencies > 1 Hz (17). These findings indicate that, in healthy people during tidal breathing, the low frequency signal typically used by commercial devices of 5 Hz is not low enough to be sensitive to the entire lung, including the small airways and lung parenchyma. Therefore, the 5 Hz signal mostly reflects the mechanics of larger, more central airways.

When oscillometry first appeared commercially, it was commonly stated that R5 reflects total respiratory system resistance, and R20 reflects large airway resistance, so R5-R20 reflects small airway resistance. This interpretation of R5-R20 is nearly universally adopted, but it is important to recognize that there is no gold standard measure of SAD (4). The view that R5-R20 is a measure of SAD is overly simplistic and does not consider frequency or time constants, nor does it capture the mechanical behavior of the lung as a complex, heterogeneous assembly of airways arranged both in series and parallel embedded in the lung parenchyma (18). Goldman rationalized R5-R20 as an indicator or peripheral airways disease by noting that, while only resistance at 0 Hz would truly reflect total respiratory resistance, resistance at 5 Hz is as low as typical commercial systems will go and therefore is a good estimate of total respiratory resistance. Then, if R20 is an indicator or central airway resistance, R5-R20 equals total minus central airway resistance, this difference being peripheral airway resistance. However, since a 5 Hz signal is missing resistance in the smaller, more peripheral airways and lung tissues, R5 is really an underestimation of total lung resistance, and R5-R20 is more accurately the difference between resistance in more central vs. more peripheral airways and not necessarily in the small airways only. Interestingly, the large ATLANTIS study, which examined multiple aspects of SAD in participants with asthma using different modalities, recognized this additional contribution of larger, peripheral airways when it defined R5-R20 as “a measure reflecting respiratory resistance of small to medium sized conductive and peripheral airways” (19).

The difference between R5 and R20 is not just a mathematical difference, but importantly reflects the frequency dependence of R, first observed in patients with obstructive lung disease (11) and subsequently in asymptomatic smokers (20). The mathematical expression of respiratory system impedance as a function of frequency reveals that only reactance is frequency dependent; resistance is independent of frequency and reflects resistance of the airways (21). The finding of frequency dependence of R, with increases in R at lower frequencies (typically < 5 Hz), indicates that R is not acting as a pure Newtonian resistance (i.e., R = Pressure/Flow), and other factors must be influencing low frequency R. These factors can be understood by considering frequency dependence of compliance (C). Frequency dependence of C had previously been recognized as a method to detect SAD, provided there was no evidence of abnormal overall lung C or of central airway obstruction (22). Frequency dependence of R has a similar interpretation, and, indeed, frequency dependence of R and C are well correlated (20). Oppenheimer and colleagues have demonstrated that frequency dependence of C correlates with R5-R20 in people with COPD or after exposure to World Trade Center dust, supporting R5-R20 as a measure of SAD (23, 24).

**Figure 1:**
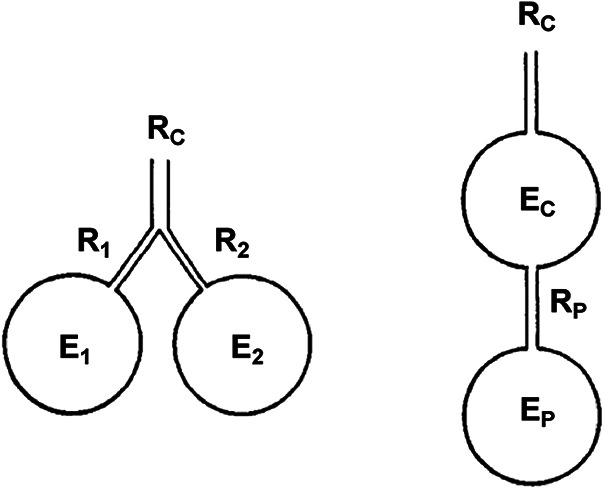
Linear compartment models of lung mechanics. Left: The parallel compartment model described by Otis and colleagues shows a central airway, characterized by central resistance (Rc) leading to 2 branching airways characterized by their respective resistances, R1 and R2. These each lead to separate parenchymal compartments characterized by their respective elastances, E1 and E2. Right: The series compartment model described by Mead shows a central airway with its resistance (Rc) and elastance (Ec) leading to a peripheral airway with its own resistance (Rp), which then leads to a terminal parenchymal compartment with its own peripheral elastance (Ep). Both models result in frequency dependence of resistance if there are any differences between R1/E1 vs. R2/E2 in the Otis model, or if Rp > Rc or Ep > Ec in the Mead model.

Understanding the mechanical determinants of frequency dependence or C or R has traditionally been explained by mathematical modeling of the respiratory system. Mathematical models are critical to understanding the physiologic meaning of oscillometry measurements, but they are models only and do not reveal the ground truth of the structure of the respiratory system. The simplest model was described by Otis and colleagues, who invoked uneven time constants (the product R x C in one airway-parenchymal unit) in parallel airways to explain frequency dependence of R or C (25). Mead demonstrated that if the airways are arranged in series, with compliant central airways leading to peripheral airway-parenchymal units, then frequency dependence of R or C can also develop if either the peripheral airway R or the central airway C increases (i.e., the time constant of the series elements becomes uneven) (26) (Figure 1). Of course, some combination of both models is more realistic in the complex, branching structure of the lung. Note that the size of airways is not specified in these models and applies to any size airway depending on how they are arranged in parallel or in series. These fundamental principles demonstrate that frequency dependence of R does not require involvement of the small airways *per se*. Instead, in the case of bronchoconstriction, heterogeneity is the key factor explaining overall changes in frequency dependence of R (27), with contribution of lung parenchymal tissue resistance at low frequencies < 1 Hz (15, 16). Nevertheless, computational modeling demonstrates that much of the heterogeneity arises in the lung periphery at the small airway level. In particular, functional lung imaging that incorporates an anatomically accurate branching tree structure along with constant-phase tissue elements reveals that at least some of the airways involved must be < 2 mm in diameter, thus involving the small airways (28, 29). But while this is a necessary criterion for frequency dependence, it does not require only small airways be involved.

If R5-R20 were solely reflecting SAD, then measuring oscillometry while breathing a mixture of helium-oxygen (heliox) should not affect R5-R20, because the lower density of heliox reduces turbulent flow, which occurs predominantly in larger, central airways. However, one study has shown that R5-R20 is decreased, at least in some participants, when breathing heliox, indicating that some turbulent flow is measured in this range and therefore implicates the presence of larger, central airways in the R5-R20 signal (30). Another study demonstrated that breathing heliox increases R5-R20 in the setting of peripheral bronchoconstriction, but mainly due to reduction in R20, not elevation in R5, which is expected since R20 primarily reflects resistance due to turbulent flow in the larger, central airways (31). These findings emphasize that R5-R20 is not specific for SAD.

Other mathematical models reveal that frequency dependence of R arises from heterogeneity in the mechanical properties of not only the arrangement of airways and parenchymal compartments but also of the lung parenchyma itself (32). One model demonstrates that frequency dependence of R is seen when there is alveolar pressure inequality, which can arise from bronchi larger than 2 mm in diameter as well as heterogeneous changes in the lung parenchyma, as might be seen in emphysema and lung fibrosis (33). Computational modeling further reveals that time-varying elastance, as might occur during recruitment and derecruitment of lung parenchyma, can also result in frequency dependence of R (34).

While frequency dependence is commonly driven by an increase in R5 greater than any change (typically increase) in R20, it can also be driven by a decrease in R20 (as in the heliox example above). The most common explanation for decreased R20 is upper airway or proximal airway shunting, where the energy of oscillation is lost or dissipated, particularly at higher impedances, at the higher frequencies that reflect the upper airways, mouth, cheeks and central airway walls (35, 36). This would result in a fall in R20 contributing to an increase in R5-R20 and frequency dependence of R. In fact, conducting airway elastance and upper airway shunting play a significant role in frequency dependence as seen by R5–20 (37). The presence of upper airway and airway wall shunting was thought to interfere with the ability of frequency dependence of R to reflect central vs. peripheral airway function (38), but frequency dependence may still useful in considering peripheral airway function, even after taking into account the effect of shunt flow in this regard (39), with the caveats discussed.

Other modeling shows X5 to better reflect heterogeneity of peripheral airway narrowing and SAD than R5-R20 (40), possibly because R5-R20 includes largely non-obstructed central airways embodied in R20 (41). Because fixed cut-offs of R5-R20 and X5 to define normality have been determined and may be confusing for clinicians to remember or use, oscillometry ratios, like R5-R20/R5 to reflect peripheral resistance and X5/AX to reflect peripheral reactance, have been proposed and perform well compared to FEV_1_/FVC in predicting poor asthma control (42). Combining multiple breath washout with oscillometry to measure the effects of methacholine on lung mechanics has suggested that changes in X (and consequently AX) are more indicative of heterogeneity, while change in R5-R20 may be better explained by changes in central airway caliber rather than heterogeneity (43). R2 alone or compared to R8 (R2-R8) may also yield insight into SAD and have the advantage of not including R20, which is typically above the resonant frequency and therefore encompasses some component of inertia, complicating its interpretation (44); however, it is not possible to reliably measure R2 with current commercial oscillometry systems.

While ventilation heterogeneity typically arises from SAD, it may also be caused by heterogeneity in larger airways and in parenchymal time constant differences (33). There are data that demonstrate that ventilation heterogeneity, assessed by imaging or MBNW, does not necessarily correlate with R5-R20 (45–48), suggesting that R5-R20 and ventilation heterogeneity likely reflect different physiologic processes related to SAD.

Despite these theoretical considerations, R5-R20 proves to be a useful measure of SAD. Recent computational modeling has demonstrated strong statistical associations between SAD by imaging and R5-R20 (49). Furthermore, ventilation heterogeneity on imaging has been associated with R5-R20 and implicates SAD (50). One study has shown direct associations between R5-R20 and small airways as assessed by optical coherence tomography (51).

Most importantly, interpreting R5-R20 as a marker of SAD has proven clinically useful. In multivariate analysis, R5-R20 and AX were associated with increased risk of future asthma exacerbations among the large ATLANTIS cohort (52). The effects of obesity on oscillometry measures relate to increases in R5-R20, AX and resonant frequency, implicating SAD in healthy, obese people as well as obese people with asthma and COPD (53), and may be linked to outcomes in patients with asthma (54). Ventilation heterogeneity detected by imaging and oscillometry both relate to quality of life and disease control in patients with obstructive lung disease (55). Detecting SAD by oscillometry in asthma can now be considered a treatable trait since it is measurable, linked mechanistically to symptoms, control and outcomes, and treatable by using small particle inhaled medications or biologic therapy (7, 56). In fact, a recent study suggested non-responders to biologic therapy in asthma had more evidence of SAD (57).

Tracking SAD by oscillometry is also useful in disease monitoring, for example in patients with premature birth (58) or interstitial lung disease (59). Monitoring lung health in children with oscillometry has proven especially valuable (58, 60, 61). Therefore, even though it is important to consider other causes of frequency dependence of R, oscillometry is a clinically useful tool to detect SAD with significant relevance for important patient outcomes.

### Dependence of oscillometry measures on lung volume

Respiratory system impedance is highly dependent on lung volume (62–67). However, currently available commercial devices do not adjust impedance for the lung volume at which tidal breathing occurs. Therefore, changes in R, for example, may not specifically reflect changes in intrinsic airway narrowing. Instead, changes in R may also reflect the external effect of parenchymal tethering on airway diameter that is due to the interdependence of airways and lung parenchyma.

This recognition of the interdependence of the airway and lung parenchyma is dealt with during body plethysmography where the reciprocal of Raw is calculated as airway conductance (Gaw) (68), and then adjusted for lung volume by dividing by the volume at which panting occurred (thoracic gas volume, TGV) to yield specific airway conductance (sGaw). Now airway resistance can be considered independently of lung volume, with sGaw reflecting changes in intrinsic airway caliber. Other measures of Raw adjusted for lung volume are also described (69).

The problem of lack of adjustment of impedance values for lung volume was recognized by Pride in 1992, who suggested multiplying R by mid-tidal lung volume to create a composite measure that accounted for the average lung volume over the full respiratory cycle during which impedance is measured (specific R, or sR) (38). Only recently has there been a study to investigate a volume-adjusted index of R (70). Specifically, the degree of ventilation heterogeneity measured by the lung clearance index (LCI) from multiple breath washout in children with cystic fibrosis was compared to R5, X5, and R5 adjusted for lung volume by taking the reciprocal of R5 divided by FRC (specific conductance at 5 Hz, or sG5) (70). Indeed, sG5 was more closely correlated and a better predictor of LCI than the volume unadjusted measures of R5 and X5. In addition, while altered lung volume as seen by high RV/TLC diminished the correlations of R5 and X5 with LCI, the correlation of sG5 with LCI was unaffected, indicating it was a more robust measure of intrinsic airway resistance (70). Thus, correcting oscillometry measures for lung volume, perhaps by using sG5, may allow for more specific detection of true airway resistance abnormalities under circumstances where lung volume might also be affected.

### Lack of disease discrimination by oscillometry

Oscillometry is a very sensitive method to detect abnormal lung function in the setting of respiratory symptoms in a general population (71, 72). But oscillometry does not necessarily perform better than spirometry, so it may best serve a complimentary function to spirometry (73). Measurements determined by oscillometry are interdependent and the overall results may lack specificity for lung disease. For example, due to lung volume effects, restrictive lung disease may manifest as an increase in X5 representing stiffer lung, but also an increase in R5 due to smaller lung size (the volume effect described above), a combination of findings that is seen similarly in obstructive lung disease and obesity (74, 75).

**Figure 2:**
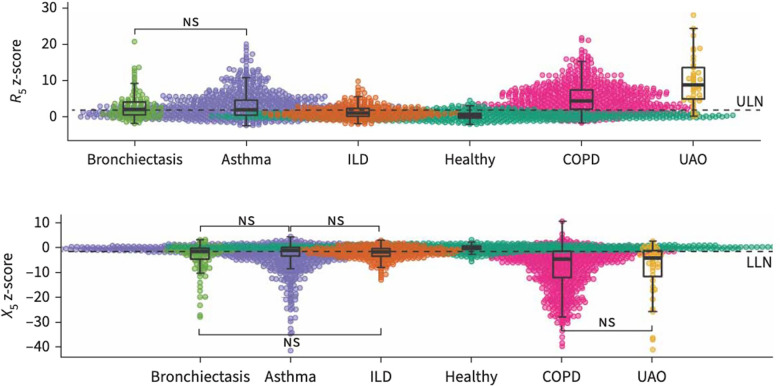
Comparison of Z-scores of R5 (top) and X5 (bottom) Among different lung disease groups. Data are median (interquartile range). Non-statistically significant comparisons are indicated (NS); all other groups are statistically different. Notice that while statistical differences are seen between some groups, there is significant overlap in values. LLN=lower limit of normal. ULN=upper limit of normal. ILD=interstitial lung disease; COPD=chronic obstructive pulmonary disease; UAO=upper airway obstruction. From Liang (54), under Creative Commons Attribution Non-Commercial License 4.0.

While few studies exist that focus on the specificity of oscillometry for lung disease, the overlap in R and X in different disease states is illustrated in the study by Liang and colleagues (76) (Figure 2). While some of these disease profiles are statistically different from each other on a population level (e.g., healthy vs. asthma, COPD, ILD, UAO) the substantial overlap seen makes oscillometry a poor test by which to distinguish different disease states in an individual. Of course, the same is true of all lung function tests including spirometry, lung volumes, diffusing capacity or multiple breath washout. All of these must be interpreted in the correct clinical context before rendering a clinical diagnosis, as is emphasized by recent ERS/ATS interpretation standards (77). Oscillometry may be useful in differentiating diseases with overlap of obstruction and restriction, such as combined pulmonary fibrosis and emphysema (CPFE) (78) or asthma-COPD overlap (79), which are necessarily best evaluated by synthesizing physiology, imaging and clinical data. Machine learning methods may be promising for enhancing the discrimination of asthma and COPD by oscillometry (80).

One way in which oscillometry can better distinguish between diseases is examination of within-breath changes, or the changes in R and X during the separate phases of inspiration and expiration. Asthma and emphysema appear to be differentiated on the basis of intrabreath changes in X5 (*Δ*X5) (81). A *Δ*X5 > 2.8 cm H_2_O/L/s is associated with expiratory flow limitation and appears to be unique to emphysema and not asthma, likely from diminished airway tethering due to emphysema that results in excess airway narrowing on expiration. Interestingly, while more negative X5 during *exhalation* identifies obstructive lung disease distinguishing between emphysema from asthma, more negative X5 during *inhalation* appears to detect restrictive lung disease, distinguishing between ILD from COPD (82–85). Here it is thought that the more negative X5 during inhalation reflects greater lung stiffness from lung expansion during inhalation in a lung with increased elastic recoil from fibrosis. Therefore, close examination of within-breath oscillometry (specifically X5) may help distinguish between obstructive and restrictive lung disease if the parenchymal changes are sufficient to affect X5.

Given that oscillometry is very sensitive for lung disease compared to spirometry, one might consider that an important role for oscillometry is determining when lung function is normal. We can gain much insight on this by examining the results of the LEAD study (72). The LEAD study found that abnormal oscillometry was found in a significant number (16.4%) of the general population of study participants who had respiratory symptoms (*n* = 2,171) but normal spirometry. This finding could then lead to further evaluation, depending on the clinical situation. Meanwhile, 9.8% of the total population (*n* = 7,560) had abnormal oscillometry with normal spirometry and no symptoms. Whether this group has subclinical disease or this finding just reflects the high sensitivity of oscillometry is unknown and will only be discovered by following such people longitudinally. Finally, 17.6% of the total population had respiratory symptoms or disease with normal spirometry and oscillometry, so it is possible that even normal oscillometry cannot rule out underlying respiratory disease.

## Conclusion

Oscillometry is a very sensitive measure of lung mechanics and may detect abnormalities even when spirometry is normal. However, it is important to appreciate limitations in interpreting oscillometry. First, frequency dependence of R does not necessarily implicate SAD only, but may also be due to heterogeneity of airway and lung mechanics, even though much of the heterogeneity likely arises in the lung periphery at the small airway level. Since interpretation of oscillometry is inferred from mathematical modeling of the respiratory system, such interpretation relies on underlying conditions of the model system and parameters. Other causes of frequency dependence of R supported by mathematical modeling include large airway and parenchymal heterogeneity, and upper airway and airway wall shunting. Second, it is important to recognize the effects of lung volume on measurement of R and X, since the interdependence of airways and lung parenchyma necessarily links the two regions mechanically, and airway diameter and extent of airway patency will vary with lung volume. Finally, while very sensitive, oscillometry is not specific for lung disease at a population level, which is similar to other lung function tests. However, patterns of R and X may provide insight into underlying lung disease, and particularly within-breath measures of X may help differentiate asthma, emphysema, and ILD. Future work to better understand disease specificity using oscillometry will require complimenting oscillometry with other measures of disease like spirometry, lung volumes, diffusing capacity, multiple breath washout, and imaging. While considering these limitations, oscillometry remains a very sensitive tool and is useful for assessing overall lung health and detecting early abnormalities even when spirometry is normal. Therefore, oscillometry provides a valuable addition to spirometry, as well as to other lung function tests, imaging, and clinical presentation, by being able to detect abnormalities of the lung periphery that involve the small airways and affect R5, R5-R20, X5 and AX. The additional physiologic information provided by oscillometry on SAD and other aspects of respiratory mechanics will contribute to confidence in making a clinical diagnosis of lung disease and monitoring lung health over time.
